# The Nursing and Care of the Sick Prior to 1850

**Published:** 1914-06-06

**Authors:** F. M. Sandwith

**Affiliations:** Gresham Professor of Physic, etc.


### THE NURSING AND CARE OF THE SICK PRIOR TO 1850.

By Dr. F. M. SANDWITH, F.R.C.P., Gresham Professor of Physic, etc.

### AN HISTORICAL ADDRESS AT GRESHAM COLLEGE.

Nursing, like every other branch of knowledge, has passed through a slow course of evolution from a very elementary attendance on the sick, practised among primitive people, to the new art and new science of nursing which is a notable feature of modern life. We could no more tolerate the old standard of nursing than we could submit to the bleeding, blistering and leeching of the physician of former generations. But this fine art in nursing is a modern product, an innovation of our own generation, a humanising factor in civilised life which only those can sufficiently appreciate who either owe their return to health and strength to the skilful ministrations of the modern nurse or who, as doctors, have felt their absolute dependence on her reliability and devotion in the treatment of a difficult case. "Upon nursing proper under scientific heads," says the greatest exponent of trained nursing, "physicians or surgeons must depend partly, perhaps mainly, whether nature succeeds or fails in her attempts to cure sickness."

Although visiting the sick must have appealed to charitable people as soon as civilisation taught them humanity and sympathy with the sufferings of others, the first institutions for the reception of patients and the first organised system of nursing known to us are due to certain Christian Orders, who extended their influence and justified their existence by this means. The members of these Orders worked under clerical supervision, and among them many noble men and women found an outlet for their religious zeal. Among the deaconesses of the early Christian Church were the wives or widows of Roman consuls and senators, and their missionary enthusiasm caused some of them to abjure the world and imitate the sanctity and dirt of the orthodox saints. Thus Chrysostom, Bishop of Constantinople, lauds the "unspeakable coarseness" of the attire of Olympia, the beautiful young wife of a Roman prefect, who "abstained from all animal food, and went for the most part unwashed."

### Nursing by Religious Orders.

From the beginning of the fifth century for upwards of a thousand years the nursing of the civilised world was almost entirely in the hands of the men and women who were bound by vows to a life of self-denial and religion, who ministered to the mind diseased even more, perhaps, than to the poor sick body. Wherever misery was greatest and disease most loathsome, there these unobtrusive heroes and heroines could be found, whilst they were exhorted not to be squeamish in the performance of the most menial office, for their labours would secure for them "an everlasting crown."

The task of nursing the sick still lies in the hands of religious sisterhoods in most Catholic countries, and on the spirit of discipline and self-denial which distinguish their Orders the best nursing societies among lay people have been founded; and though their want of scientific training has, unfortunately, caused the religious sisters to be displaced in many instances by the hospital-trained nurse, yet the high ideals and truly Christian spirit in which they carry on their ministrations should be an example to the highly trained woman, who occasionally loses her tenderness for the patient in her intellectual interest in the case. The title "Sister," which distinguished the more responsible nurses in hospitals, is, of course, a survival of the religious Orders, where the superior female workers become Sisters of Charity.

Although most of the old hospitals were the offshoots of monasteries, one still survives which was founded by private munificence, the Hôtel-Dieu in Paris, built and endowed by Bishop Landry in the year 600 A.D. It is now a hospital for the sick only, but it was originally a hospice for the needy of every description. The attendance on the inmates was rendered by men and women of religious Orders, and it was under the ecclesiastical management of the Chapter of Notre-Dame. In 1327 two surgeons were appointed to visit the sick daily, and others studied medicine in the wards, of whom Ambroise Paré may be mentioned as a distinguished student and teacher.

Were I to show an illustration on the lantern of a ward in this famous hospital, drawn in the sixteenth century, you would realise the religious atmosphere which predominated. The spiritual needs of the patients were probably better understood by the excellent sisters than the art of nursing, for in the picture you will see that each bed generally contained two patients, and several attendants are engaged in religious exercises, whilst the King is kneeling in the foreground. From the annals of the hospital we find that complaints were made of the overcrowding prevalent; in 1690 it is stated that five or six adults or nine or ten children were put into a single bed, "which causes the death of many." A century later the state of overcrowding was worse than ever, dead bodies were sometimes left in the wards for one or more days, the air was pestilential, the least wound became gangrenous; and although, as it was said, a young surgeon might there learn the art of operating and dressing, "the way of curing wounds he cannot learn. Every patient he takes in hand (do what he will) must die of gangrene." The well-known surgeon Petit urged the rebuilding of the hospital outside Paris, but the sisters would not tolerate such an innovation, and they prevailed.

These sisters, of the strict Order of St. Augustine, nursed the sick for upwards of a thousand years in this hospital. Their methods were crude and absolutely unprogressive, but their devotion was magnificent. What would be said now of work such as is described thus: "The sisters endured with cheerfulness and without repugnance the stench, the filth and the infections of the sick, so insupportable to others that no other form of penitence could be compared with this species of martyrdom. No one who saw the religious sisters of the Hôtel-Dieu not only do dressings, make beds, and bathe patients, but also in cold winter weather break the ice in the River Seine and stand knee-deep in the water to wash the filthy clothes, could regard them as other than holy victims, who, from excess of love and charity for their neighbours, hastened willingly to the death which they courted amidst the stenches and infections"? But, as time went on, devotion alone could not suffice, and paid nurses were introduced to supplement the nursing done by the sisters, for new methods of doctoring called for newer methods of nursing, and the narrow education of the nuns, which was entirely religious and non-scientific, had to give place to a more modern type of nurse.

### What was Doing in England.

England somewhat lagged behind the Continent in the building of hospitals, and the first one we hear of is an establishment for the reception of persons suffering from the King's evil, founded by the Archbishop of Canterbury in 1070. Next comes the great hospital, St. Bartholomew's, built by Rahere, jester to Henry I., about 1123. He had grown tired of fooling and entered a religious Order, but he persuaded his old master, the King, to grant him an empty space in the western suburbs of the city, the Smoothfields, now called Smithfield, where he built a priory and hospital, the sick being nursed by members of the Order. In front of the hospital was the space where jousts and tournaments were held, and the brethren of the hospital doubtless had the privilege of attending those who were injured at these sports.

Next to St. Bartholomew's comes St. Thomas's, which was founded on the site of the Convent of St. Mary Overie in Southwark, where the present London Bridge Station stands. It was meant originally as an Almery for "indigent children and necessitous proselytes," but afterwards became a hospital for the aged and infirm.

But here, as in the Hospital of St. Bartholomew, grave abuses arose under their ecclesiastical governorship, and when King Henry VIII. confiscated the monastic possessions, including the hospitals, the act was generally considered justifiable by the citizens. But if the suppression of the religious Orders was no unmixed evil, the misery and destitution of the sick was soon felt to be terrible, and was deemed by the citizens to be so intolerable that they petitioned the King to allow the reopening of certain hospitals. In 1544 St. Bartholomew's was re-established on a secular basis, a matron and twelve sisters being appointed to nurse the sick. The duty of the matron was to receive the patients from the hospitaller and put them "in such convenient places within this house as you shall think fit." She had charge of all the sheets, blankets, and bedding, and kept the sisters under strict discipline, not allowing them to associate with anyone outside the hospital, and they were enjoined to "avoid and shun the conversation and company of all men." Besides attending on the sick they had to clean the wards, spin the flax, and wash the patients' clothes.

The citizens of London contributed generously to the reopening of the hospitals, and in this reign, and in that of Edward VI., systematic attempts were made to establish institutions to fulfil those duties which the religious Orders had undertaken for so many centuries.

### Early Red Cross Work.

Nursing the sick and wounded in time of war and stress was the duty and privilege of many noble women, and in civilised countries such mercy was extended to the fallen enemy as well as to the victim in his own country; but when the need for such assistance arose in far-distant lands the pity of the inhabitants could often not be reckoned on, and there arose some famous Orders of Military Hospitallers. The one of which we have the fullest details, and which survives in modified form to this day, is the Order of St. John of Jerusalem, and the story of its foundation is worth recording.

From the very dawn of Christianity pilgrims, with scrip and staff, sallied forth from distant countries to visit the places in Palestine made holy by the great Founder of our faith, and for many years the perils attending such a journey were not overwhelming. But when strife began among pagans and Christians (and later Muhammedans), for the possession of Jerusalem, the pilgrimage became one of great difficulty, though the dangers only added to the glory of its achievement and the pilgrimages continued unabated. At last, in 1050, certain rich merchants established in Jerusalem two hospitals, one for men under the protection of St. John, and one for women under that of St. Mary Magdalene. Here the poor of every nation, and even the Muhammedans themselves, were relieved. "Those whom robbers had plundered were re-clothed, those whom disease had debilitated were tended with skill and tenderness, and those who died were buried with Christian rites. This hospital of the Almoner was the cradle of the illustrious fraternity, the Knights Hospitallers of St. John of Jerusalem, of Rhodes and of Malta."

In time the stories of the cruelties and sacrileges inflicted by the Turks so aroused the wrath of Christendom that men banded themselves together to fight for the Cross, and for several centuries sick Crusaders filled the Hospital of St. John, where they were tended by the brethren of the Order. Many warriors laid down their arms and devoted their lives in gratitude to help others as they had been helped, and the Order was richly endowed by benefactors from many countries. The government of the hospital was at first secular, but after some years the brothers and sisters formed themselves into a religious fraternity under the rule of St. Augustine. As their wealth increased their work became more extended and other hospitals were established, and the brothers, we read, "never left the apartments of the sick but to give themselves up to prayer, or to take the field against the enemies of the Cross" (Vertot).

The hospitallers were of three kinds, knights, priests, and serving brothers, who all devoted themselves to the sick and were often members of great families and nobles. Their number, at one time, was said to be no less than 30,000. They founded a large hospital in Malta in 1575, which still stands and which filled a need for many years. The Order, which was originally founded for nursing the sick, ceased to devote itself exclusively to this duty as time went on, and though the English Order was re-established early last century, its duties have somewhat changed, but it is still familiar to most of us by its work, the St. John Ambulance Association, St. John's Brigade nurses, and as hospital orderlies and bearers. The Order still controls the ophthalmic hospital in Jerusalem.

### The Rise of Secular Societies.

With the slow decadence of monastic Orders, including in many instances those devoted to the care of the sick, there arose outside the Church many secular societies devoted to this purpose, but even these were apt to come, sooner or later, under the control of the clergy. Among such secular orders, the greatest and most enduring is, perhaps, that of St. Vincent de Paul. Of good peasant stock, this splendid man, gifted with boundless charity and excellent common-sense, passed through an adventurous youth and then obtained permission to assist in the nursing of the poor in the Hospital of La Charité in Paris.

Anxious to secure the help of the wealthy for the needs of the poor without embarking on ill-regulated charity, he persuaded certain ladies of Châtillon to form themselves into an Association of Charity in 1617, for the purpose of visiting those poor whose cases had been examined, giving them food and ministering to their souls as well as to their bodies, besides giving them burial if they died. Presently he formed a men's branch for the suppression of professional begging, he instituted night refuges, farm colonies and workshops. Not satisfied with this work, he devoted himself to the rescue and relief of the unfortunate galley slaves, and in order to assist the escape of one of them he actually took his place and served as a slave until his identity became known.

A Ladies' Association (Dames de Charité), which he originally started in Paris for visiting the sick in hospitals and providing them with extra comforts, developed in a short time into a vast organisation under the influence of its founder, so that nursing became a fashionable pursuit, and by 1633 the work was being carried on all over Europe. The Order trained nurses for visiting the sick in their homes, and these Sisters of Charity were not cloistered nuns, but moved freely in the world, though they were not of it. They have worked in great cities and in country districts, on battlefields and through the French Revolution, and when we come to the story of the Crimea we shall find that England was called upon to wonder and envy the efficiency of the military hospitals of our French allies, where the Sisters of St. Vincent de Paul had gone in vast numbers to tend the sick and wounded.

### Hospital Regulations in Sixteenth Century.

In spite of the efforts of the citizens in London and elsewhere in England, the substitution of secular institutions for the care of the sick and needy in the place of religious Orders resulted in a great accession of hardship to the poor, and English nursing was left far behind that which was carried on by religious sisters on the Continent.

Nursing was no longer the expression of a religious calling, and patients were no longer treated with intense devotion and self-sacrifice, prompted by Christian ardour, nor with the discipline exacted by the Orders; their fate was delivered into the hands of hirelings, who might be devoted, but who often were not, whose service was of a very menial kind, and whose supervision was left to ill-paid and uneducated women little better than themselves. No wonder, therefore, that the art of nursing sank to a condition of neglect which resulted in unspeakable misery to the sick, in deterioration of the hospitals and, one can have little doubt, in the neglect and death of innumerable people. It is of interest to read the regulations laid down in the sixteenth century in a big hospital for the guidance of matron and nurses :—

"Ye shall faithfully and charitably serve and help the poor in all their griefs and diseases . . . and, above all things, see that ye avoid, abhor and detest scoldings and drunkenness as most pestilent and filthy vices." Again, the rules enact that the attendants "give the medicines as directed; the night medicines by eight o'clock in winter and nine in summer," and that "they appoint some sober patient to crave a blessing and return thanks at every meal." The nurse must also "scour and make clean the beds and floors of the whole ward, with the tables and forms, the passage and stairs, and garrets. . . . She must attend the butler at the ringing of the beer bell and take with her such patients as are able to carry the beer in safety to the ward, and not suffer such patients to waste or embezzle it by the way."

But, in spite of regulations and calls to their better feelings, the status of the nurse sank lower and lower until it reached its greatest depth of degradation by the middle of the nineteenth century.

### What John Howard Reported.

John Howard, the famous philanthropist, has left us a description of various hospitals at home and abroad which he visited towards the end of the eighteenth century, their condition and, incidentally, the state of the nursing. Although he was a strict Calvinist himself, he speaks in the highest praise of the devotion to the sick shown by friars and nuns. "The great attention of the nuns distinguish the hospitals in Roman Catholic countries," he says. But he criticises the foul air, the absence of ventilation and want of cleanliness in some hospitals, as in the Hospital of St. John of Jerusalem at Malta, where it was found necessary to perfume the miserable patients to hide their "noxious odour," and the physicians kept a handkerchief before their noses when they approached the bedside; whilst the nuns were pronounced to be "the most dirty, ragged, unfeeling and inhuman persons."

When he speaks of British hospitals and infirmaries it makes sad reading. Of all the London hospitals he only recommended Guy's as being clean and well ordered; in the London Hospital he noted "in a dirty room in the cellar there is a cold and hot bath, which seem seldom used." At the Westminster Hospital "a sum is paid every year for the destruction of bugs and blankets. A very sickly boy had not his clothes taken off for a fortnight. Two lay in the bathing tub . . . the felons in the gaols were better accommodated as to cleanliness and bedding, and their cells were less offensive."

Of an infirmary in Ireland he says: "In two of the rooms . . . there were thirteen beds and fifteen patients. In a room called the tower two patients and a little dirty hay on the floor, on which, they said, the nurse lay . . . no sheets in the house and the blankets very dirty; no vault, no water. The surgery was a closet about ten feet six inches, its outfit consisting of ten vials, some of them without corks, a little salve stuck on a board, and some tow. Here I saw one naked pale object who was under the necessity of tearing his shirt for bandages for his fractured thigh."

### At the End of the Eighteenth Century.

In the very last years of the eighteenth century, it may be mentioned that one of the officials at Guy's Hospital was still the "bug catcher," who received a salary of £40, the same as that given to a surgeon. "I am persuaded," wrote Howard, "that much depends on the patients lying on fresh and clean beds . . . if the annual sum spent in several hospitals for the destruction of bugs was expended in airing, beating, and brushing the beds, the end would be much better answered."

But what hope was there for the hospitals when nursing had sunk to the low ebb at which it was then and for many years after? Only women of the lowest class entered the profession, and as late as 1857 a defence, written by one who signed himself "One who has walked a good many hospitals," shows the kind of treatment to which they were submitted.

"Hospital nurses have been much abused," he writes; "they have their faults, but most of them are due to the want of proper treatment. Lectured by committees, preached at by chaplains, scowled at by treasurers and stewards, scolded by matrons, sworn at by surgeons, bullied by dressers, grumbled at and abused by patients, insulted if old and ill-favoured, talked flippantly to if middle-aged and good-humoured, tempted and seduced if young and well-looking—they are what any woman might be under the circumstances."

### Conditions at Haslar.

Here, again, are some of the rules laid down for the nurses at Haslar Royal Naval Hospital, which give a graphic picture of the squalor and dirt present: "That no dirt, bones or rags be thrown out of any window, or down the bogs, but carried to the places appointed for that purpose; nor are any clothes of the patients, or others, to be hung out of any of the windows of the house. That any person concealing the escape of any patient from her ward . . . be discharged the hospital. That all nurses who disobey the matron's orders, get drunk, neglect their patients, quarrel or fight with any other nurses, or quarrel with the men . . . be immediately discharged."

The answer to an inquiry made early in the nineteenth century regarding the qualifications of nurses is instructive: "They always engage them without any character, as no respectable person would undertake so disagreeable an office. He says the duties they have to perform are most unpleasant, and that it is little wonder that many of them drink. The only conditions that are made are . . . that they are not confirmed drunkards."

### What Charles Dickens Wrote.

We are all acquainted with our old friend Mrs. Gamp, from whom I propose to quote a few samples in the art of nursing, for there is no doubt that Charles Dickens, with his masterly pen, drove home to the people of England the fact that the British nurse was a blot on our social system; the admirable portrait of this villainous old woman helped largely towards the extermination of her kind. Nor must it be supposed that this was merely a gross caricature, for Forster, in his Life of Charles Dickens, says:—

"In his preface to the book he speaks of her as a fair representation, at the time it was published, of the hired attendant on the poor in sickness; but he might have added that the rich were no better off, for Mrs. Gamp's original was in reality a person hired by a most distinguished friend of his own, a lady, to take charge of an invalid very dear to her; and the common habit of this nurse in the sick room, among other Gampish peculiarities, was to rub her nose along the top of the tall fender. . . . The portentous Mrs. Gamp, with her grim grotesqueness, her filthy habits and foul enjoyments, her thick and damp but most amazing utterances, her moist clammy functions, her pattens, her bonnet, her bundle, and her umbrella. This world-famous personage has passed into and become one with the language, which her own parts of speech have certainly not exalted or refined."

"Every vice was rampant among these women," says Sir Henry Burdett, "and their aid to the dying was to remove pillows and bed clothes, and so hasten the end." But whilst some public-spirited individuals now arose who made gallant efforts to reform the art of nursing, it must in fairness be said that some nurses were much better than others. Sir James Paget, surgeon to St. Bartholomew's Hospital, wrote thus of them, in an address on "St. Bartholomew's Hospital and School fifty years ago," which he delivered in 1885:—

### Address by Sir James Paget to the Abernethian Society on Nursing in 1830.

". . . In the department of nursing there is the greatest and happiest contrast of all. It is true that even fifty years ago there were some excellent nurses, especially among the sisters in the medical wards, where everything was more gentle and orderly than in the surgical. There was an admirable Sister Hope, who had had her leg amputated in the hospital, and then spent her life in giving others the most kindly, watchful care. A Sister Mary, a near relative of hers, was as constant to her charge, and there were some good surgical sisters too. They had none of the modern art; they could not have kept a chart or skilfully taken a temperature, but they had an admirable sagacity and a sort of rough practical knowledge which were nearly as good as any acquired skill. An old Sister Rahere was the chief among them, stout, ruddy, positive, very watchful. She once taught an erring house-surgeon where and how to compress a posterior tibial artery; she could always report correctly the progress of a case, and from her wages she saved all she could and left it in legacy to the hospital. And there was her neighbour, Sister Colston, rough tongued, scolding, not seldom rather tipsy, and yet very watchful and really very helpful, especially in what she felt to be good cases. On the whole, indeed, it may fairly be said that the sisters were among the very best nurses of the time. The ordinary nurses were not so; the greater part of them were rough, dull, unobservant, and untaught women; of the best it could only be said that they were kindly, and careful, and attentive in doing what they were told to do.

"Nursing was not at that time, nor had it ever been, a subject of careful study, either needing or encouraging intelligence, and I think that in this country, since its separation (except in the abiding name of Sister) from the tasks of the members of religious Orders, it had scarcely been regarded as a work of mercy or of philanthropy. It was not till twenty years later, in the Crimean War, that Miss Nightingale showed what might be done in hospitals by highly cultivated, courageous and benevolent gentlewomen; and the noble example which she showed had, I think, more influence than anything else that can be told of in the production of the happy changes in the midst of which you work.

"I believe, indeed, that in 1830 the admission of young ladies to be nurses in this or any similar hospital could not have been seriously proposed. It would have been called indecent, audacious, unprincipled, and I know not what besides; the notion of their being associated with medical students would have been deemed utterly vile; nothing but vile mischief would have been foretold of it. But I believe as fully that no more mischief would have come then than comes now, and that is none. Young ladies were quite as sensitive, as prudent, and as virtuous then as they are now, and the students were as respectful to them in their own homes and in society. If they had chosen to work in hospitals they would have been as safe from insult or annoyance as they were in the ballroom. Really, the question of admitting young ladies to learn nursing was never raised, because none at that time wished to learn it. There was far less earnest love of any of the kinds of active benevolence which you see now; much less prevalent religious zeal; very little district visiting or teaching of the poor; very little desire for the exercise of practical knowledge anywhere but at home. And so ladies did not wish to learn nursing either for the sake of charity, or in the love of useful work, or the weariness of an unoccupied life.

"When they did begin to wish it they were too much and too long hindered. It was thought rather bold when a lady was admitted to attend my lectures; the courtesy of the students of that time showed that at least one could be safe. I wish it had been believed that I might have lectured then with as much propriety as I hope I do now to the hundred who are here this evening."

### German Manuals of Nursing from 1825.

To appreciate the instruction at that time considered valuable to a nurse, it is well to study some of the manuals of nursing published early in the nineteenth century, mostly by German physicians, because the Germans, as we shall see in continuing the history of nursing, were at that time far in advance of the English in their methods and in the type of woman who undertook nursing. Fresh air, especially night air, was considered dangerous to an invalid, so that it must never be admitted except indirectly, through an adjoining room, though, for the sake of deodorising a sick room, the window might be thrown open twice a day with great precautions against chill. Elaborate directions are given for burning various substances to conceal the close smells by others less unpleasant. "Vinegar should be kept constantly boiling," says an English book on nursing; "the steam impregnates the air with fine antiputrescent vapour." Washing a patient from head to foot, which every nurse now considers a daily necessity except in cases of extreme illness, was then unheard of, but a nurse is advised to wash the patient's hands and face daily! In all directions for nurses it is obvious that no high educational standard, no great powers of observation, no technical skill nor intelligence were expected or asked for. A nurse must, as the text-books insist, be honest, sober and obedient, and at the best she was drawn from the domestic class; at the worst, as generally in England, from a much lower order.

A little book entitled "The Good Nurse," printed in 1825, has lately fallen into my hands. It is not so much a manual of nursing as a tract, for besides giving some practical hints on nursing, it gives excellent advice to husbands not to become too fond of their clubs during the illness of their wives, and to wives not to become so absorbed in worldly gaieties as to neglect their families. It includes numerous and often startling prescriptions for medicines which the nurse is advised to administer freely, and on her own responsibility, to the patient, and when hints are given for the management of the nurse's own health we read advice such as the following :—

"A dish of strong coffee should be brought to her first thing in the morning; a glass of wine should be given as the occasion may require, and a little brandy when anything occurs which affects the bystander, such as moving a patient in an infectious disease or on opening a broken limb. Infection is taken in a moment. I recommend one ounce of best bark to be put into a quart of spring water, the rind of a Seville orange, and a quarter of a pint of good brandy, shaking it well and taking a glass frequently. It is our imperious duty to pay a proper regard to self-preservation." A nurse is advised not to take snuff, as being an objectionable habit. "This unprincipled conduct," the book says, "I know is frequently practised in the sick chamber; the medicines are put aside, the patient being disgusted with their smell or taste, and nurse fancies it a part of her business to comply in imposing on the physician by stating that they have been administered." Finally, "a large bottle of vinegar, one of water, and another of brandy and port wine" should always be in the sick room, "although they may not be wanting for the patient, yet they may be very necessary for those attending in the room."

### An Appeal of 1847.

It did not, apparently, occur to women of good social standing that they might find a mission worthy of their highest efforts in tending the sick, except, perhaps, in their own families. A very touching appeal was issued in 1847 by Sir Edward Parry, then Superintendent of the Royal Naval Hospital at Haslar, calling upon the women of England to come forward, as many were doing in Germany, to undergo a course of training and then assist in the nursing of their countrymen in hospital. "Their duties," he admits, "will be arduous and self-denying, and they will meet with much to exercise their Christian patience and forbearance, but they will receive encouragement and support from the captain superintendent and the other officers." But the time was not ripe for the spirit of self-sacrifice to break down the prudish, narrow views of the early Victorian woman, trained to elegance but not to usefulness. Not one woman came forward to offer her services; his appeal fell on absolutely deaf ears. A more dramatic appeal to their emotions, and, above all, the example of a commanding genius able to break down all prejudice and opposition, were needed to show the way to the women of England.

